# Somatostatin 2 Receptors in the Spinal Cord Tonically Restrain Thermogenic, Cardiac and Other Sympathetic Outflows

**DOI:** 10.3389/fnins.2019.00121

**Published:** 2019-02-20

**Authors:** Belinda R. Bowman, Phillip Bokiniec, Simon McMullan, Ann K. Goodchild, Peter G.R. Burke

**Affiliations:** ^1^Department of Biomedical Sciences, Faculty of Medicine and Health Sciences, Macquarie University, Sydney, NSW, Australia; ^2^Max Delbrück Center for Molecular Medicine, Berlin, Germany; ^3^Neuroscience Research Australia, Sydney, NSW, Australia

**Keywords:** somatostatin, spinal cord, autonomic, sympathetic, brown adipose tissue temperature

## Abstract

The anatomical and functional characterization of somatostatin (SST) and somatostatin receptors (SSTRs) within the spinal cord have been focused in the dorsal horn, specifically in relation to sensory afferent processing. However, SST is also present within the intermediolateral cell column (IML), which contains sympathetic preganglionic neurons (SPN). We investigated the distribution of SSTR2 within the thoracic spinal cord and show that SSTR2A and SSTR2B are expressed in the dorsal horn and on SPN and non-SPN in or near the IML. The effects of activating spinal SSTR and SSTR2 were sympathoinhibition, hypotension, bradycardia, as well as decreases in interscapular brown adipose tissue temperature and expired CO_2_, in keeping with the well-described inhibitory effects of activating SSTR receptors. These data indicate that spinal SST can decrease sympathetic, cardiovascular and thermogenic activities. Unexpectedly blockade of SSTR2 revealed that SST tonically mantains sympathetic, cardiovascular and thermogenic functions, as activity in all measured parameters increased. In addition, high doses of two antagonists evoked biphasic responses in sympathetic and cardiovascular outflows where the initial excitatory effects were followed by profound but transient falls in sympathetic nerve activity, heart rate and blood pressure. These latter effects, together with our findings that SSTR2A are expressed on GABAergic, presumed interneurons, are consistent with the idea that SST2R tonically influence a diffuse spinal GABAergic network that regulates the sympathetic cardiovascular outflow. As described here and elsewhere the source of tonically released spinal SST may be of intra- and/or supra-spinal origin.

## Introduction

Neuropeptides encoded by about 70 genes influence neuronal activity within the central nervous system (Burbach, 2010). The inhibitory neuropeptide somatostatin (SST) is distributed widely throughout the CNS, in its two biologically active forms, SST-14 and SST-28. Within the spinal cord SST immunoreactive terminals are present in the dorsal horn which receives primary sensory information and the intermediolateral cell column (IML), the major source of sympathetic preganglionic neurons (SPN) (Krukoff, 1987; Patel, 1999; Schulz et al., 2000). SSTR1-4 are present in the spinal cord (Segond von Banchet et al., 1999; Schulz et al., 2000). The spinal distribution of SST and somatostatin receptor (SSTR) suggests involvement in modifying afferent information entering the dorsal horn and the activity of the SPN. Although effects at the dorsal horn are well established (Kuraishi et al., 1985; Sandkühler et al., 1990; Shi et al., 2014; Takahashi et al., 2014), there has been no investigation into the role SST exerts at the sympathetic outflow in the thoracic spinal cord that may modify cardiovascular and/or metabolic activity.

Somatostatin modulates cardiovascular and metabolic functions at higher levels of the neuraxis (Burke et al., 2008; Cote-Vélez et al., 2017). For example, inhibiting ventral medullary nuclei with SST results in apneusis, bradycardia, hypotension sympathoinhibition, and attenuation of chemo- and somatosympathetic reflexes (Burke et al., 2008, 2010) and these effects were blocked using selective SSTR2 antagonists (Burke et al., 2008). Furthermore all premotor sympathetic cell groups contain SST including those in the ventral medulla (Millhorn et al., 1987; Strack et al., 1989a), serotonergic neurons of the raphe (Loewy and McKellar, 1981; Chiba and Masuko, 1989), the A5 cell group (Strack et al., 1989a) and neurons in the paraventricular nucleus of the hypothalamus (PVN) (Sawchenko and Swanson, 1982). These data further suggest that SST in the spinal cord influences sympathetic function.

We explored this hypothesis. Our initial targets were SSTR1 and SSTR2 as these are the most abundant SSTR in rodents (Gunther et al., 2018), however, as SSTR1 is found presynaptically, acting often as an autoreceptor, we focused our attention on SSTR2. We identified the distribution of SSTR2A and SSTR2B-like immunoreactivity within the thoracic spinal cord and showed expression in the dorsal horn and on SPN together with an association of SSTR2A with GABAergic neurons. We investigated the functional effects of SST and an SSTR2 agonist applied intrathecally to the upper thoracic spinal cord on sympathetic, cardiovascular and thermogenic outflows and determined the functional consequences of blocking SSTR2. Our data suggest that, within the thoracic spinal cord, SST acts on SSTR2 expressing sympathetic and dorsal horn neurons to tonically suppress sympathetic activity and interscapular brown adipose tissue (iBAT) thermogenesis. Additionally, we propose that SST tonically suppresses, via SSTR2, a spinal network of GABAergic neurons that modulates sympathetic and cardiovascular outputs.

## Materials and Methods

Adult, male, Sprague Dawley rats (450–550 g; Animal Resources Centre, Perth, Australia) were used in accordance with the guidelines of the Australian Code of Practice for the Care and Use of Animals for Scientific Purposes and all procedures were approved by the Animal Care and Ethics Committee and Biosafety Committee, of Macquarie University.

### Anatomical Experiments

#### Immunohistochemistry

Immunohistochemistry was conducted as described previously (Bowman et al., 2013; Parker et al., 2013). Briefly, pentobarbitone anesthetized rats (80 mg/kg, *n* = 4) were perfused transcardially with saline followed by 4% paraformaldehyde (PFA) in phosphate buffer (pH 7.4, fixative). Spinal cords (C8-T3 and T4-T10), placed in fixative overnight, then sectioned parasagittally or coronally (50 μm) using a vibrating microtome. Free-floating sections were washed 3 × 30 min in TPBS (Tris-HCl 10 mM, sodium phosphate buffer 10 mM, 0.9% NaCl, pH 7.4) then incubated 48 h at 4°C in TPBS with 0.01% merthiolate (TPBSm) containing 5% normal horse serum and primary antibodies to detect SPN (goat anti-choline acetyl transferase [ChAT], 1:800, #AB144P, RRID:AB_2079751, Millipore, United States) and SSTR2A (1:100, #SS-800, RRID:AB_2491103, Biotrend, Germany) or SSTR2B receptor (1:750, #SS-810, Biotrend, Germany). After three washes, sections were incubated overnight at 4°C in TPBSm containing 2% normal horse serum and secondary antisera: Alexa Fluor^®^ 488-conjugated donkey anti-goat (#A-11055, RRID:AB_2534102, Invitrogen, Australia) and Cy3-conjugated donkey anti-rabbit (#711-165-152, RRID:AB_2307443, Jackson ImmunoResearch, United States). Sections were washed, mounted with Fluoromount^TM^ mounting medium (#K024, Diagnostic Biosystems, Pleasanton, CA, United States) and coverslipped when almost dry.

#### Retrograde Tracing

Retrograde tracing was carried out as described previously (Bowman and Goodchild, 2015) with the tissue used here acquired from the animals used in this previous study. Rats (*n* = 12) were anesthetized with ketamine (75 mg/kg) and medetomidine (0.5 mg/kg, ip). Cephazolin (200 mg, im) and carprofen (2.5 mg/kg sc) were administered. Animals were secured in a stereotaxic frame and a laminectomy exposed T2. Cholera toxin B (CTB, 1% 2 × 100 nl injections, #103C, List Biologicals, United States) was bilaterally microinjected into the spinal cord centered on the lateral horn. The skin wound was closed, povidine-iodine (Betadine, Faulding Pharmaceuticals, Australia) applied to the area and the animal was administered atipamezole (1 mg/kg sc) and monitored closely.

After 2 days, rats were reanesthetized with sodium pentobarbital (80 mg/kg ip), perfused as described above and brain and spinal cord were removed and placed fixative overnight before processing. CTB injection sites within the spinal cord were shown previously (Bowman and Goodchild, 2015).

#### Combined Immunohistochemistry and *in situ* Hybridization

Combined immunohistochemistry and *in situ* hybridization was carried out on brains and spinal cords cut coronally using a microtome (VT1200S, Leica, Wetzlar, Germany; 40 and 100 μm, respectively). Spinal cord injection sites were identified using a modified nickel intensified diaminobenzidine (DAB) reaction as described and demonstrated previously (Bowman and Goodchild, 2015).

In brain sections fluorescent immunohistochemical detection of CTB was conducted using a rabbit anti-CTB primary antibody (1:5,000, #7927, RRID:AB_2313635, ViroStat, Portland, ME, United States) in conjunction with detection of mRNA using digoxigenin (DIG)-labeled riboprobes as described previously (Kumar et al., 2010; Bowman et al., 2013; Bowman and Goodchild, 2015). Sense (forward) and antisense (reverse) riboprobes for preprosomatostatin (PPS) were designed as previously published (Burke et al., 2008), with a T7 promoter attached to the 5′ end of the antisense primer and an SP6 promoter attached to the sense primer as follows (promoters in uppercase):

**Forward:** GGATCCATTTAGGTGACACTATAGAAGctcaagctcggctgtctgag
**Reverse:** GAATTCTAATACGACTCACTATAGGGAGAggaggagagggatcagaggt

Detection of GAD67 mRNA in SSTR2A expressing neurons of the spinal cord was conducted as described above, in 40 μm thick parasagittal or coronal sections of spinal cord segments C8-T3 and T4-T10. A primary antibody raised in rabbit was used to target SSTR2A (#SS-800, Biotrend, Germany), and a donkey anti-rabbit secondary antibody (Cy3-conjugated, #711-165-152, Jackson ImmunoResearch, United States) for visualization. The following sense and antisense primers were designed for GAD67 riboprobe (promoters in uppercase) as described previously (Burke et al., 2008; Bowman et al., 2013):

**Forward:** GGATCCATTTAGGTGACACTATAGAAGttatgtcaatgcaaccgc
**Reverse:** GAATTCTAATACGACTCACTATAGGGAGAcccaacctctctatttcctc

#### Imaging and Analysis

All imaging was conducted using an AxioImager Z1 (Carl Zeiss, Germany). Spinal cord sections were scanned for ChAT-ir neurons within the lateral horn or close to the central canal. ChAT-ir nests were imaged and quantified in C8-T3 sections, by counting the number of ChAT-immunoreactive (-ir) cells which expressed SSTR2A in each animal. Results were expressed as mean percentage of ChAT-ir cells expressing SSTR2A ± SEM.

The presence of CTB-ir and PPS mRNA expression were identified in brain sections containing the presympathetic cell groups: the caudal raphe (pallidus, obscurus and magnus), RVMM (paragigantocellular and parapyramidal groups), RVLM, A5 region and PVN. After scanning the rostrocaudal extent of each premotor group, the Bregma level which had the highest expression of CTB was identified. One or two additional sections were also selected for inclusion in analysis, according to the longitudinal spread of CTB labeling within a cell group. Thus cell groups with a longer rostrocaudal extent of CTB-ir were allocated three Bregma levels for analysis, while those with a smaller rostrocaudal CTB-ir distribution were allocated two levels. Sections from the following Bregma levels were analyzed for double labeling: the raphe was analyzed at -13.90, -12.12 and -11.52 mm; the RVMM analyzed at -13.56, -12.96 and -12.24 mm; the RVLM analyzed at -12.36 and -12.00 mm; the A5 region analyzed at -10.20 and -9.72 mm and the PVN analyzed at -2.04 and -1.72 mm. Cells were counted bilaterally, and the number of CTB-ir neurons expressing PPS mRNA was determined. Results were expressed as the average percent (±SEM) of retrogradely labeled cells expressing PPS in each region.

### Physiological Experiments

#### Surgical Preparation

Surgical preparation was conducted as described previously (Burke et al., 2008, 2010). Rats were anesthetized with urethane (ip, 10% in saline 1.3 g/kg) and core temperature was monitored via flexible rectal thermistor and maintained (36.5–37°C) using a heating blanket (Harvard Apparatus, Holliston, MA, United States). Thirty minutes before intrathecal injection, core temperature was adjusted to 36°C to generate brown adipose tissue thermogenesis, as previously described (Tupone et al., 2011). The right femoral artery and jugular vein were cannulated for measurement of arterial pressure and administration of drugs and saline, respectively. Animals were vagotomized and intubated for artificial oxygen-enriched ventilation (rodent respirator #7025, UGO Basile, Italy), and secured in a stereotaxic frame. Rats were paralyzed with pancuronium bromide [0.8 mg iv, followed by an infusion (0.1 mg/ml in saline) at 2 ml/h]. End-tidal CO_2_ (Capstar-100, CWE Inc., PA, United States) was maintained between 3.5 and 4.5% under control conditions.

Heart rate was calculated using the R wave of ECG recordings. Recordings of efferent sympathetic nerve activity (SNA) were made from the cut, left greater splanchnic nerve using bipolar silver wire electrodes. Signals were amplified (BMA-400, #09-03010, CWE Inc., United States), band pass filtered (0.1 Hz–3 kHz) and digitized for recording using Spike2 software (Cambridge Electronic Design Ltd., Cambridge, England). Data were normalized for comparison by taking the activity present at the nadir following euthanasia (see below) as zero nerve activity and 100% nerve activity was taken as the average of activity 5 min prior to intrathecal drug injection. The temperature of left and right iBAT deposits was measured using T2 thermocouple probes (#BT-1, Physitemp, Clifton, NJ, United States) attached to a T-type Pod (#ML312 & #ML305, ADInstruments, Sydney, Australia) connected to a PowerLab (30 series, ADInstruments, Sydney, Australia) then digitized for recording. Thus, three sympathetic outflows were monitored: HR (vagotomised), splanchnic SNA and iBAT temperature.

In two animals the adrenal glands were removed bilaterally via the retroperitoneal cavity and the adrenal blood vessels ligated.

#### Intrathecal Drug Administration

Dura beneath the atlanto-occipital membrane was incised, and a catheter (od 0.61; id 0.28 mm) inserted in the sub-arachnoid space and advanced to spinal level T5–T6, as described previously (Bowman and Goodchild, 2015).

Intrathecal injections of drug (agonists (SST 1 mM [#H-1490.0005, Auspep, Australia], seglitide 1 mM [#S1316, Sigma Aldrich, United States]) and antagonists (BIM-23627 (45 μM, 150 μM, 450 μM and 1.5 mM) [#H-5886.1000, Bachem, Switzerland], CYN-154806 1 mM [D Tyr form, #1843, Tocris, United Kingdom])) or vehicle (saline or phosphate buffered saline, PBS, pH 7.4) were conducted using a Hamilton syringe. Five microliter of drug or vehicle, flushed with 7 μl of vehicle, was injected over 10–15 s. MAP, HR, splanchnic SNA, iBAT temperature, end tidal CO_2_ and core temperature were recorded for at least 60 min following injection. The concentration of SST was the same as used previously (Burke et al., 2008).

The animals were then euthanized with 3 M KCl (0.3 ml iv), and the level of nerve activity recorded, and a laminectomy was performed to confirm the position of the catheter tip.

#### Data Analysis

Electrophysiological data were analyzed as the peak change from baseline (pre-injection) values. Data are presented as the mean ± the SEM. GraphPad Prism and/or SPSS (Statistical Package for the Social Sciences) were used for statistical analysis and the data were considered significant at *p* < 0.05. Where peak changes due to drug were compared to vehicle, Student’s *t*-test was used to compare responses. Where the effects of the different doses of antagonist were compared, mean peak and trough responses across treatment groups were analyzed separately via analysis of variance (ANOVA). Dunnett’s *t*-test adjustments were used to compare individual responses to control (saline). *A priori* polynomial contrasts were used to assess systematic trends (linear, quadratic, etc.) across treatment means for the main dependent variable.

## Results

### SSTR2 Are Densely Expressed in the Dorsal Horn, on SPN and on GABAergic Neurons in the Thoracic Spinal Cord

**FIGURE 1:**
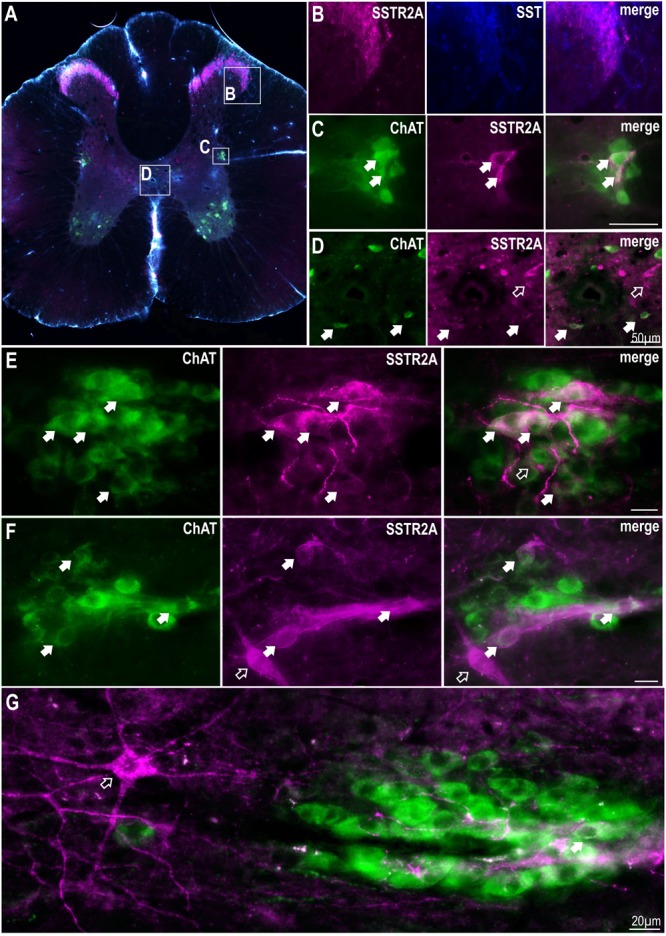
SSTR2A are expressed in dorsal horn, on SPN and non-ChAT labeled neurons in the thoracic spinal cord. **(A)** Coronal section of thoracic spinal cord showing distribution of SSTR2A-like (magenta), SST (blue) and ChAT immunolabeling (green). **(B)** Dorsal horn showing overlapping SSTR2-like and SST labeling. **(C)** Intermediolateral cell column (IML) containing ChAT labeled SPN with some exhibiting SSTR2A-like immunoreactivity (filled arrows). **(D)** Central autonomic area containing some SSTR2A expressing ChAT labeled SPN (filled arrows). **(E–G)** “Nests” of ChAT labeled SPN some of which co-express SSTR2A-like labeling (filled arrows). Scale bars = 20 μm. Open arrows indicate non-ChAT SSTR2A labeled neurons close to or adjacent to SPN.

Although SST terminals have been described around SPN no SSTR have been described here thus, we sought to identify the location of SSTR2A and SSTR2B in thoracic spinal cord. Dense immunolabeling of SSTR2A and SST was found in lamina II of the dorsal horn (Figure 1A,B) as has been previously described (Schulz et al., 1998; Todd, 2017). Some, but not all, ChAT labeled SPN in the IML and around the central canal expressed SSTR2A-like immunoreactivity (Figure 1C–F). About 10% of SPN in the IML expressed SSTR2A-like immunoreactivity in C8-T3 spinal cord segments (10.1 ± 1.2% of 582 SPN counted, *n* = 3) with some nests having multiple SSTR2A expressing neurons (Figure 1E,F) but many having none. Non-ChAT labeled neurons both close to, and more distant, from the IML, also expressed SSTR2A-like immunoreactivity (Figure 1F,G).

**FIGURE 2:**
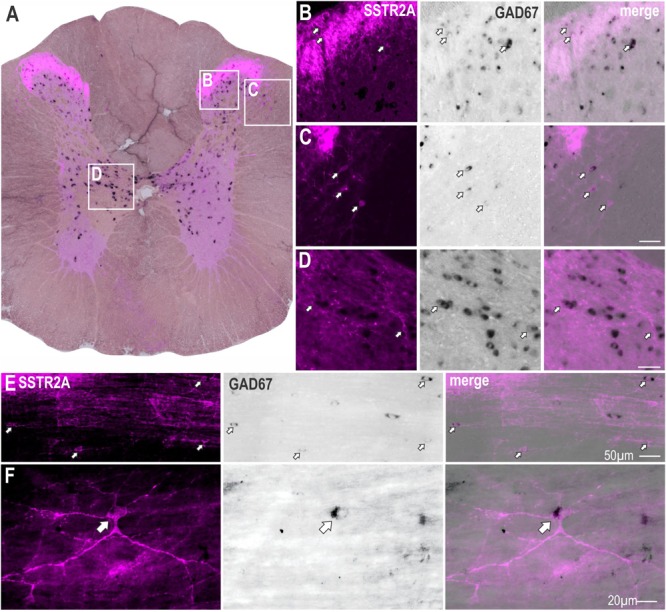
Some SSTR2A expression colocalises with GAD67 mRNA in thoracic spinal cord. **(A)** Coronal section of spinal cord showing SSTR2A-like immunoreactivity (magenta) and GAD67 mRNA (black). Some neurons in dorsal horn **(B)**, dorsolateral funiculus **(C)**, central autonomic area **(D)** and lamina III **(E)** co-express both markers. **(F)** Neuron adjacent to IML expressing SSTR2A-like immunoreactivity and GAD67 mRNA. Filled arrows indicate double-labeled neurons. Scale bars for B–E = 50 μm and for F = 20 μm.

To determine whether non-ChAT SSTR2A positive neurons were GABAergic, *in situ* hybridization for GAD67 mRNA was combined with SSTR2A immunolabeling (Figure 2A). SSTR2A-like immunoreactivity was co-expressed with GAD67 mRNA in some neurons in the dorsal horn, as previously described (Todd, 2017) (Figure 2B) including lamina III (Figure 2E), the dorsolateral funiculus (Figure 2C), adjacent to central canal (Figure 2D), as well as in some star shaped neurons close to the IML (Figure 2F).

**FIGURE 3:**
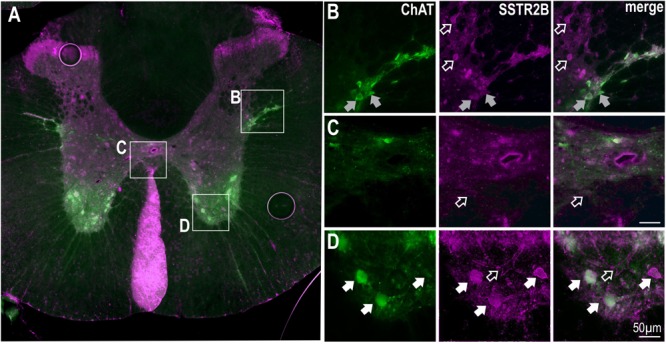
SSTR2B in dorsal and ventral horns and diffusely in the IML in the thoracic spinal cord. **(A)** Coronal section of thoracic cord showing SSTR2B-like (magenta) and ChAT (green) immunoreactivity. Clear membrane associated SSTR2B-like labeling is present dorsal and ventral **(D)** horns with diffuse labeling in intermediolateral cell column **(B)** and central autonomic areas **(C)**. Filled arrows indicate double-labeled neurons (white indicates SSTR2B-like immunoreactivity appears membrane-bound, gray indicates diffuse SSTR immunoreactivity). Scale bars = 50 μm.

SSTR2B immunolabeling was also present in lamina II of the dorsal horn and in the ventral horn (Figure 3) as previously described (Schulz et al., 1998). More diffuse SSTR2B immunolabeling was observed in other parts of the gray matter [as previously described (Schulz et al., 1998)] with some ChAT labeled SPN expressing SSTR2B-like immunoreactivity (Figure 3B). Accurate quantification was not possible due to the diffuse nature of the SSTR2B expression as previously described (Schulz et al., 1998). ChAT positive motoneurons in the ventral horn clearly expressed SSTR2B-like immunoreactivity (Figure 3A,D) but not SSTR2A-like immunoreactivity (see Figure 1A).

Thus, immunolabeling for SSTR2 is present in the spinal cord: in the dorsal horn (SSTR2A & SSTR2B) and ventral horn (SSTR2B); on some GABAergic, likely, interneurons and on SPN (SSTR2A & SSTR2B), with the latter suggesting their activation influences sympathetic outputs.

### PPS mRNA Is Found in all Sympathetic Premotor Cell Groups

**FIGURE 4:**
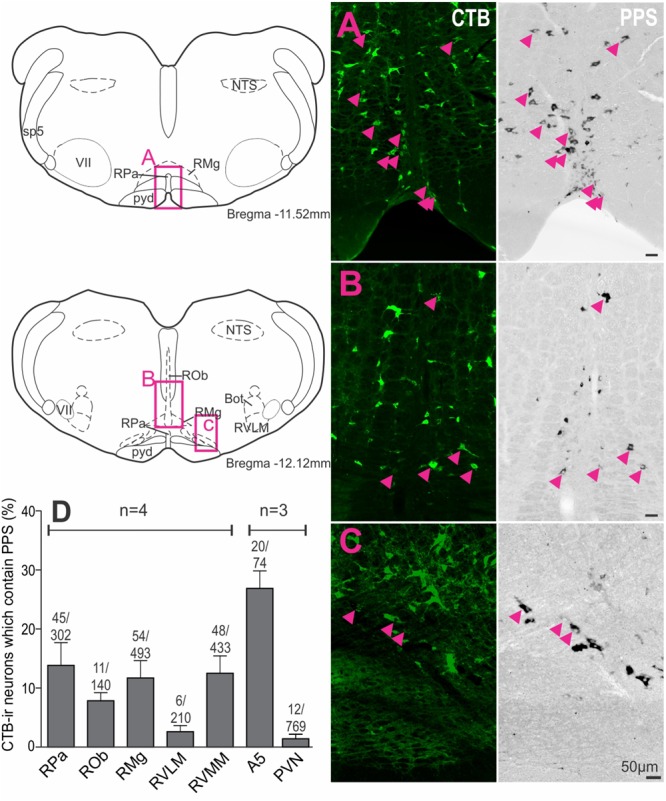
Premotor sympathetic neurons express preprosomatostatin (PPS) mRNA. **(A–C)** Premotor sympathetic regions (magenta boxes) showing CTB-ir neurons retrogradely labeled from the spinal cord (green) and PPS mRNA expression (black). Double-labeled cells are indicated by arrowheads. **(A)** Raphe pallidus (RPa) and raphe magnus (RMg), **(B)** Raphe obscurus (ROb) and raphe magnus (RMg), **(C)** Rostral ventromedial medulla (RVMM). Scale bars = 50 μm. **(D)** Percentage of CTB immunoreactive cells containing PPS mRNA within each presympathetic region examined. Data are shown as mean ± SEM. Cell counts per region are shown above each bar. Schematic diagrams adapted from Paxinos and Watson (2005).

To assess supraspinal sources of SST that could directly influence SPN, premotor sympathetic cell groups were examined to determine if they expressed PPS mRNA (*n* = 3–4). Examples of double labeling in the raphe pallidus (Figure 4A), raphe magnus (Figure 4B) and RVMM (Figure 4C) reflect the larger proportion of CTB-labeled cells double labeled for PPS mRNA in these regions (Figure 4D): raphe pallidus (13.9 ± 3.8% of 302 CTB-ir neurons), raphe magnus, (11.7 ± 2.9% of 493 CTB-ir neurons), raphe obscurus (7.9 ± 1.4% of 140 CTB-ir neurons), the RVMM (12.5 ± 3.0% of 433 CTB-ir neurons) and the region containing the A5 group (26.9 ± 3.0% CTB-ir of 74 neurons), compared to the RVLM (2.6 ± 1.0% of 210 CTB-ir neurons) and the PVN (1.4 ± 0.7% of 769 CTB-ir neurons).

Thus, all major premotor sympathetic cell groups have the potential to release SST at SPN with the greatest innervation arising from the brainstem. The predominant SST projection, representing 81% of all PPS mRNA + CTB-ir neurons, arises from the midline raphe/RVMM regions of the brainstem.

### SST or Selective Activation of SSTR2 in the Thoracic Spinal Cord Reduces Cardiovascular, Sympathetic and Thermogenic Activity

**FIGURE 5:**
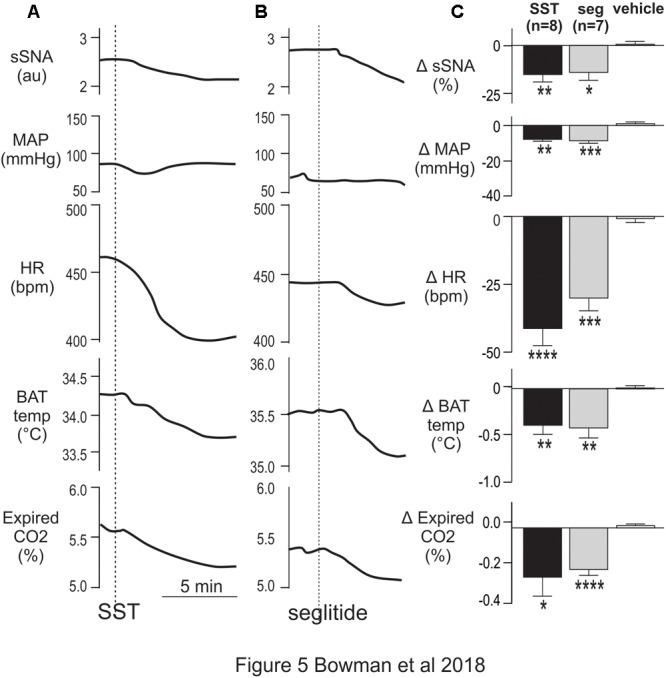
Intrathecal SST and SSTR2 agonist decrease sympathetic, cardiovascular and thermogenic outflows. **(A)** Representative responses show that SST (1 mM) elicited decreases in sSNA, MAP, HR, iBAT temperature and expired CO_2_. **(B)** The SSTR2 agonist seglitide evoked similar effects to SST. **(C)** Grouped data showing peak responses evoked by intrathecal SST (*n* = 7), seglitide (*n* = 8) or vehicle (*n* = 8). Data are expressed as mean ± SEM. Effect of each drug compared to vehicle ^∗^ = *p* < 0.05, ^∗∗^ = *p* < 0.01, ^∗∗∗^ = *p* < 0.001, ^∗∗∗∗^ = *p* < 0.0001.

As some SPN expressed SST2R-like immunoreactivity and potentially receive SST input from raphe/RVMM regions the effects of intrathecal administration of SST (total volume 5 μl, *n* = 8), at a dose previously used within the ventral medulla to reduce autonomic outflows [1 mM; (Burke et al., 2008)], was determined. Sympathetic, cardiovascular and thermogenic parameters were decreased (Figure 5A). SST evoked significant sympathoinhibition (-15.1 ± 3.8 vs -0.3 ± 1.1%, *t*_(8)_ = 3.71; *p* = 0.006), modest hypotension (-7.9 ± 1 vs -0.1 ± 1.0 mmHg, *t*_(10)_ = 5.38; *p* = 0.0003), and bradycardia (-41 ± 6 vs 0.5 ± 2 bpm, *t*_(14)_ = 6.32; *p* < 0.0001), compared to vehicle control (Figure 5A,C). Similarly, metabolic outflows were reduced (Figure 5A,C), with a peak decrease of -0.38 ± 0.11°C in iBAT temperature and -0.24 ± 0.09% in expired CO_2_ compared to vehicle (iBAT = 0.06 ± 0.05°C, *t*_(6)_ = 4.14; *p* = 0.0061, and expired CO_2_ = 0.00 ± 0.01%, *t*_(6)_ = 2.61; *p* = 0.04, respectively). Note that core temperature was maintained at 36° ensuring iBAT thermogenesis was active (Tupone et al., 2011). Administration of 45 μM SST (*n* = 2) elicited a smaller bradycardic response (-19 bpm) but did not alter any other parameter measured (data not shown). PBS used as a vehicle control did not change any parameters measured, as previously observed (Bowman and Goodchild, 2015).

To determine whether SSTR2 contributed to the responses observed, the SSTR2 selective agonist Seglitide was administered intrathecally (5 μl, 1 mM, *n* = 7) and decreased cardiovascular and thermogenic outflows measured (Figure 5B,C). As observed with SST, application of Seglitide resulted in sympathoinhibition (-14.0 ± 4.1 vs -2.54 ± 0.77%; *t*_(12)_ = 2.73; *p* = 0.018), bradycardia (-30 ± 4.7 vs -2.6 ± 1.2 bpm; *t*_(12)_ = 5.68; *p* = 0.0001) and hypotension (-8.7 ± 1.5 vs 1.3 ± 1.4 mmHg; *t*_(12)_ = 4.85; *p* = 0.0004) when compared to vehicle. Moreover, decreases in iBAT temperature (-0.41 ± 0.10 vs 0.01 ± 0.03°C; *t*_(6)_ = 3.91; *p* = 0.0079) and expired CO_2_ (-0.20 ± 0.03 vs 0.001 ± 0.01%; *t*_(12)_ = 6.61; *p* < 0.0001) also resulted from selective SSTR2 activation.

These data indicate that activation of SSTR, and specifically SSTR2, in the spinal cord reduces sympathetic, cardiovascular and thermogenic parameters.

### Blockade of SSTR2 in the Spinal Cord Increases Sympathetic, Cardiovascular and Thermogenic Parameters

We then sought to determine whether SSTR2 expressing spinal neurons tonically influence sympathetic, cardiovascular or thermogenic activity.

**FIGURE 6:**
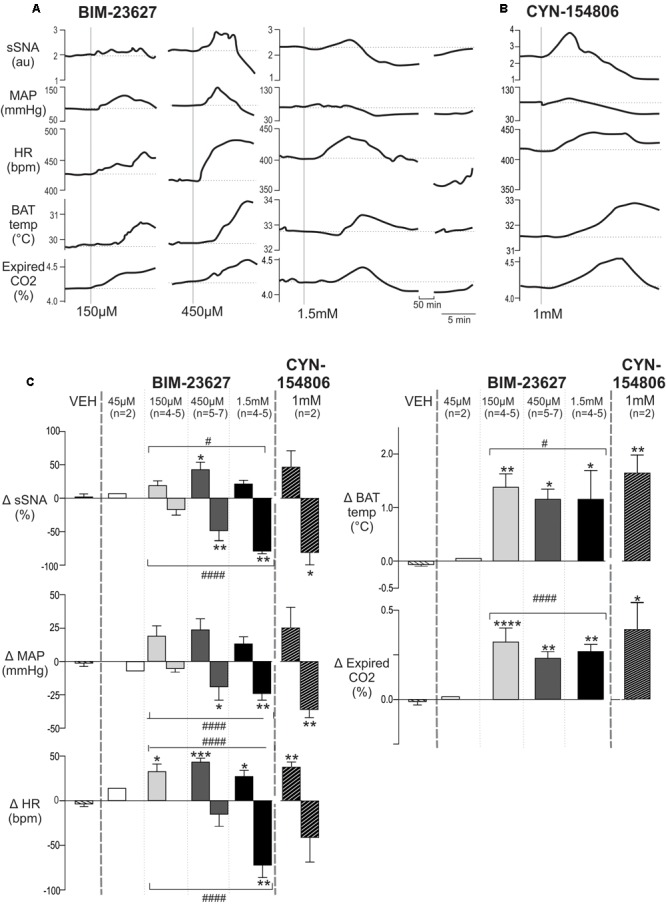
Effects of intrathecal SSTR2 antagonists on sympathetic, cardiovascular and thermogenic outflows. **(A)** Representative responses show that 150 and 450 μM of the SSTR2 antagonist BIM-23627 evoked increases in sSNA, MAP, HR, iBAT temperature and expired CO_2_. Higher doses (1.5 mM) of BIM-23627 elicited smaller early increases in sSNA, MAP, HR, iBAT temperature and expired CO_2_ followed immediately by large falls below baseline in sSNA, MAP and HR. IBAT temperature also declined but not below baseline. **(B)** CYN-154806 (1 mM) evoked similar effects to BIM-23627. **(C)** Grouped data showing peak changes relative to baseline with little to no effect seen with vehicle or 45 μM BIM-23627 (*n* = 2). In contrast 150 or 450 μM BIM-23627 evoked large early increases in all parameters. Biphasic responses in SNA, MAP and HR were evident at doses greater than 150 μM BIM-23627. All doses of BIM-23627 (except 45 μM) and CYN-154806 increased iBAT temperature and expired CO_2_. Data are expressed mean ± SEM, asterisks denote significant difference compared to PBS control (^∗^ = *p* < 0.05, ^∗∗^ = *p* < 0.01, ^∗∗∗^ = *p* < 0.001, ^∗∗∗∗^ = *p* < 0.0001), and the octothorp denotes either significant linear or quadratic trends (# = *p* < 0.05, #### = *p* < 0.0001).

Figure 6A shows representative responses to increasing doses of the peptide antagonist BIM-23627 which exhibits high affinity for SSTR2 (Tulipano et al., 2002). A low dose of 45 μM BIM-23627 had little effect on any parameter (data not shown) whereas 150 μM BIM-23627 increased sympathetic, cardiovascular (HR, MAP) and thermogenic (iBAT, expired CO_2_) parameters with 450 μM amplifying these effects (Figure 6A). Higher concentrations of BIM-23627 (450 μM and 1.5 mM) produced a biphasic response, where the initial increase in sympathetic and cardiovascular outflows was followed by a secondary decrease in these parameters (Figure 6A).

Very similar effects in all parameters were evoked by CYN-154806 (1 mM) (Figure 6B), also a peptide antagonist with a high affinity for SST2R. Importantly with both antagonists all parameters returned to baseline within ∼75 min.

The dose response grouped data are shown in Figure 6C, with the peak increase and subsequent nadir shown for each dose of BIM-23627 [45 μM *n* = 2 (not shown), 150 μM *n* = 4–5, 450 μM *n* = 5–7, 1.5 mM *n* = 4–5] and for CYN-154806 (1 mM, *n* = 3). These were compared to the largest change evoked by vehicle (VEH).

In relation to the peak sympathetic and cardiovascular responses ANOVA revealed significant dose-dependent effects on sSNA and HR (*F*_(3,14)_ = 3.44, *p* = 0.044, and *F*_(3,18)_ = 9.03, *p* = 0.0007, respectively), but not MAP (*F*_(3,14)_ = 2.58, *p* = 0.095). A significant quadratic effect was observed on peak sSNA (*F*_(1,15)_ = 6.43, *p* < 0.05) and HR (*F*_(1,18)_ = 17.53, *p* < 0.0005), suggesting that selective SSTR2 inhibition increased cardiovascular outflows before decreasing them. The maximum increase occurred at 450 μM BIM-23627, where the sympathoexcitatory (42.5 ± 11.3 vs 0.70 ± 3.30%, *p* < 0.05, *n* = 5), and tachycardic (43.2 ± 4.5 vs 3.6 ± 3.0 bpm, *p* < 0.001, *n* = 7) responses peaked, before returning toward baseline. Significant tachycardia was also observed at the other doses (150 μmM; 32.5 ± 8.5 bpm, 1.5 mM; 27.0 ± 6.9 bpm, *p* < 0.05, *n* = 5 and 6, respectively).

With respect to the secondary decreased responses, ANOVA revealed significant dose-dependent effects on sSNA (*F*_(3,14)_ = 11.84, *p* = 0.0004), MAP (*F*_(3,14)_ = 4.972, *p* = 0.012), and HR (*F*_(3,15)_ = 8.24, *p* = 0.0018). In contrast to the initial peaks, a linear effect was seen in the secondary trough responses of sSNA (*F*_(3,14)_ = 36.843, *p* < 0.0001), HR (*F*_(1,14)_ = 20.49, *p* < 0.0001), and MAP (*F*_(1,14)_ = 14.67, *p* < 0.005), suggesting that more SSTR2 were influenced or were impacted for longer, resulting in significant cardiovascular decline. Significant sympathoinhibition and hypotension were evoked by 450 μM and 1.5 mM BIM-23627 (sSNA: -49.15 ± 13.0 and -79.72 ± 3.10%, respectively, *p* < 0.01, MAP: -22.7 ± 7.7 and -24.0 ± 4.3 mmHg, respectively, *p* < 0.01), whereas heart rate reached significance only at 1.5 mM (-71.7 ± 14.0 bpm, *p* < 0.01), when compared to control.

With respect to thermogenesis, peak increases in iBAT temperature and expired CO_2_ appear saturated at 150 μM BIM-23627 when compared to control (iBAT: 1.38 ± 0.25 vs -0.06 ± 0.03°C, *p* < 0.01, *n* = 4; expired CO_2_: 0.32 ± 0.07 vs -0.02 ± 0.03%, *p* < 0.0001, *n* = 5, respectively), as increasing doses of 450 μM (iBAT: 1.17 ± 0.16°C, *p* = 0.01, *n* = 5; expired CO_2_: 0.24 ± 0.03%, *p* < 0.01, *n* = 7, respectively) and 1.5 mM (iBAT: 1.16 ± 0.48°C, *p* < 0.05; *n* = 4, expired CO_2_: 0.27 ± 0.04%, *n* = 4, *p* < 0.01) did not significantly alter the peak responses reached. Only peak increases were measured for iBAT and expired CO_2_ as later changes could arise because of evoked hemodynamic alterations.

To further corroborate SSTR2 involvement in setting sympathetic, cardiovascular and thermogenic tone, the effect of CYN-154806 (1 mM, *n* = 3) was determined (Figure 6C). Although CYN-154806 evoked initial sympathoexcitatory and hypertensive effects, these did not reach significance. The sympathoinhibition and hypotension that followed, however, reached significance when compared to control (sSNA: -80.8 ± 18.6%, *p* < 0.05, MAP: -35.8 ± 6.03 mmHg, *p* < 0.01, respectively). CYN-154806 administration also resulted in significant tachycardia (37.3 ± 5.7 bpm, *p* < 0.01), increased iBAT temperature (1.64 ± 0.34°C, *p* < 0.01), and increased expired CO_2_ (0.38 ± 0.19%, *p* < 0.05) when compared to control.

In order to ascertain that the tachycardic and thermogenic effects of BIM-23627 were not due to activation of SPN innervating the adrenal gland resulting in catecholamine release potentially activating adrenergic receptors that regulate the heart and iBAT (Cannon and Nedergaard, 2004) the adrenal glands were removed in two experiments. The effect of intrathecal BIM-23627 at 1 mM was identical to the effects seen without adrenalectomy (data not shown).

## Discussion

We demonstrate that SST or SSTR2 agonist injected intrathecally in thoracic spinal cord, modulates sympathetic, cardiovascular and iBAT thermogenic tone. SSTR2-like immunoreactivity was localized to subsets of SPN, dorsal horn neurons and inhibitory interneurons. Importantly, we show that SSTR2 antagonists increase sympathetic, cardiac and iBAT thermogenic tone suggesting that SST, tonically activating SST2R, in the thoracic cord is required for the maintenance of sympathetic, cardiovascular and thermogenic tone (at least in the anesthetized preparation used here). SST potentially released from the RVMM and raphe premotor nuclei may contribute to this.

Our data confirms and extends previous studies exploring the distribution of SSTR2 in the spinal cord (Todd et al., 1998; Schulz et al., 2000). Novel data reveal that SSTR2A-like immunoreactivity and possibly SSTR2B-like immunoreactivity were present on a subset of SPN, in line with the description of SST terminals around SPN (Johansson et al., 1984; Krukoff, 1987). These data strongly support the idea that SST, released at SSTR2, reduces sympathetic activity. Intrathecal injection of SST, or the SSTR2 selective agonist Seglitide, did evoke sympathoinhibition, hypotension, bradycardia, and decreased iBAT temperature and expired CO_2_. These effects are in keeping with the well-described inhibitory effects of SST on neurons (Bou Farah et al., 2016; Gunther et al., 2018). We also found PPS mRNA in subsets of sympathetic premotor neurons in all major loci, predominantly from the midline raphe and RVMM regions. This also corroborates earlier studies describing SST-like immunoreactivity in the medullary raphe, RVMM and A5 noradrenergic neurons (Strack et al., 1989b; Jansen et al., 1995). Taken together these data suggest it is possible that inhibitory SST is released from sympathetic premotor neurons (thought to be primarily excitatory) directly at SPN.

Surprisingly, we have demonstrated that SST acts tonically at SSTR2 in the spinal cord, as intrathecal administration of two selective SSTR2 antagonists evoked sympathoexcitation, tachycardia, thermogenesis and an increase in end tidal CO_2_. Administration of high doses of both antagonists produced biphasic responses, where initial excitatory responses in all outflows were followed by rapid and sustained decreases in sympathetic and cardiovascular outflows before returning to baseline. Several mechanisms are possible.

It is plausible that the SSTR2 antagonist induced excitatory responses are evoked by removal of inhibitory SST directly at the different pools of SPN regulating splanchnic SNA, the heart and iBAT. Our data and that of others demonstrate that SST terminals are present in the IML and that there are premotor sympathetic and spinal (Jung et al., 2008) sources of SST that may be tonically released.

It is also possible that the SSTR2 antagonist evoked increases in HR, iBAT temperature and expired CO_2_ may be coordinated, as low doses of BIM-23627 induced significant and large effects in only these parameters and, such a response may represent thermogenesis, as described previously (Morrison and Madden, 2014). This may be produced directly at SPN altering both HR and iBAT temperature, however, it is likely that SSTR2 blockade prevents the actions of SST tonically released in the dorsal horn, which occurs even when no peripheral afferent stimulus is applied (Morton et al., 1988). Thus, it is possible that temperature sensitive peripheral inputs to dorsal horn neurons are gated by SST released intraspinally and, when removed thermogenesis occurs. The spinoparabrachial cold defense pathway (Morrison and Madden, 2014) may be disinhibited by SSTR2 antagonists as although SSTR2A are expressed on inhibitory dorsal horn neurons (Todd et al., 1998) a spinoparabrachial population of excitatory SSTR2A expressing neurons has been described (Cameron et al., 2015).

The increase in splanchnic SNA generated by SSTR2 blockade is unlikely related to thermogenesis as the effective antagonist doses do not align and innervation of splanchnic and BAT preganglionic neurons are independent. Therefore, a sympathetic pathway driving functions activated by the splanchnic nerve appears to be tonically inhibited by SST. As the splanchnic nerve controls both vasomotor and gastrointestinal functions this action may contribute to the increase in MAP that also occurs following SST2R blockade.

Drug movement when applied intrathecally has been assessed (Yaksh and Rudy, 1976; Nishio et al., 1989) and it is clear that spinal cord regions impacted are influenced by catheter placement, drug-type, concentration, speed of application and time. Significant sympathoinhibition, hypotension and bradycardia followed the early excitatory responses when higher doses of SSTR2 antagonists were administered. These robust responses recover within about 70 min, indicating a non-neurotoxic effect. While there is some evidence for constitutive activity in SST receptors *in vitro* (Ben-Shlomo et al., 2007, 2009) there is no evidence that the antagonists used here block constitutive activity. Studies well suited to detecting inverse agonists (i.e., GTPyS) do not report effects of the antagonists, so constitutive activation of SSTR is a formal possibility, however, available evidence is more consistent with reversal of endogenous SST tone (Gunther et al., 2018). Both peptide antagonists used here act competitively with high binding affinity at SSTR2 (Bass et al., 1996; Feniuk et al., 2000; Tulipano et al., 2002) and although previous data suggest an interaction with opioid receptors (Ghosh et al., 1997), CYN-154806 does not block μ-opioid mediated inhibition (Feniuk et al., 2000), indicating that the observed effects are due to blockade of SSTR2. The effects are consistent with the hypothesis that increasing blockade of SSTR2 results in potent disinhibition by recruiting a spinal GABAergic network that counteracts the enhanced sympathetic and cardiovascular function. The delayed effect and a higher dose of antagonists may be required to access and disinhibit such a diffuse network, as blockade at more than a few neurons in the network may be needed to drive sympathoinhibition. A tonic GABAergic network is present in the spinal cord regulating sympathetic and cardiovascular functions (Deuchars et al., 2005; Goodchild et al., 2008; Bowman and Goodchild, 2015) possibly via α5 GABA-A receptors (Wang et al., 2008) and perhaps, this is regulated by SST. In keeping with this idea GABAergic interneurons are responsible for about half (Llewellyn-Smith, 2002) of the GABAergic boutons which comprise about 50% of the innervation of SPN (Llewellyn-Smith et al., 1995) and as demonstrated here and elsewhere (Todd, 2017) GABAergic interneurons express SSTR2A. As primary afferents only contribute about 40% of SST even in the dorsal horn (Morton et al., 1989) the remainder arises from local intraspinal (Proudlock et al., 1993) or supraspinal sources as described here.

## Conclusion

The findings demonstrate that SSTR2-like immunoreactivity is present on SPN, GABAergic interneurons and dorsal horn neurons of the thoracic spinal cord and that exogenously applied SST, or an SSTR2 agonist, reduce sympathetic, cardiovascular and thermogenic activity. Blockade of endogenous SST signaling, using selective SSTR2 antagonists, increased all parameters measured with higher doses of antagonists then robustly reducing sympathetic and cardiovascular parameters. Thus, acting at multiple sites within the spinal cord, SST appears to act as a “brake,” tonically inhibiting sympathetic and iBAT thermogenic tone via SSTR2. The data are also consistent with the notion that SST tonically modulates, via SSTR2, a diffuse GABAergic network that impacts sympathetic and cardiovascular function with the source/s of spinal SST having supra- and/or intra-spinal origins.

## Author Contributions

BB, AG, and PGB conceived and designed the study. BB and PGB acquired the data. BB, PB, and AG analyzed the data. All authors (BB, PB, SM, AG, and PGB) contributed to the interpretation of the data, preparation of the figures and writing and or revising of the manuscript.

## Conflict of Interest Statement

The authors declare that the research was conducted in the absence of any commercial or financial relationships that could be construed as a potential conflict of interest.
